# Percutaneous Atrio-Ventricular Valve Interventions: Contemporary Advances and Remaining Challenges

**DOI:** 10.3390/jcm11164801

**Published:** 2022-08-17

**Authors:** Leor Perl, Ran Kornowski

**Affiliations:** 1 Cardiovascular Department, Rabin Medical Center, Beilinson Hospital, Petah-Tikva 4941492, Israel; 2 The Faculty of Medicine, Tel-Aviv University, Tel-Aviv 6997801, Israel

These are exciting exploratory times for structural/valvular heart interventions. The growth of medical knowledge is expanding, and novel therapeutic options are constantly emerging. Mitral regurgitation (MR) is the most prevalent valvular heart disease [1,2,3,4]. MR has an incidence of less than 1% before age 54 years but increases each decade, reaching more than 9% after age 75 years [4]. MR is the second most referred etiology for surgical or transcatheter intervention, only surpassed by aortic valve disease. A recent European survey revealed that MR comprised 21% of all referrals for valve interventions [5], a rate that is likely to rise following the recent publications demonstrating efficacy in mortality and morbidity reduction in patients with functional MR [6,7,8]. Tricuspid regurgitation (TR) is also a very common valvular disorder, and it becomes more common with age. According to the Framingham Heart Study, in the elderly (>70 years), a significant TR (≥moderate) was present in 1.5% of male and 5.6% of female patients, respectively [9]. It was estimated that 2.7% of older individuals have moderate–severe TR in the UK [10]. Considering the expected demographic changes, a near doubling of the population >65 years of age will take place by 2050 [11]. However, TR has remained “forgotten” until recent years, with limited therapeutic options [12,13].

In the most recent guidelines [14], percutaneous treatment of MR has become accepted for both primary MR, in case of contraindications for surgery or high operative risk, and in selected patients with severe secondary MR fulfilling the COAPT trial inclusion criteria. As for TR, percutaneous therapy may be considered in “symptomatic, inoperable, anatomically eligible patients in whom symptomatic or prognostic improvement can be expected” [14]. However, many questions remain regarding the correct pre-procedural assessment of patients, the optimal timing of the procedure, and the outcomes of unique patient populations treated by different percutaneous methods.

In this Special Issue, we have investigated some of these matters. Medvedovsky et al. explored the impact of percutaneous MR repair (PMR) during acute decompensated heart failure (ADHF). In the study, the researchers compared high-risk patients who underwent PMR during hospitalization due to ADHF with elective patients. From a cohort of 237 patients, 46 patients (19.4%) presented with severe MR of either functional or degenerative etiology who underwent emergent PMR during index hospitalization. These patients were at a higher risk for surgery compared with elective patients. While the thirty-day mortality rate was higher in ADHF patients as compared to the elective group (10.9% vs. 3.1%, respectively, *p* = 0.042), the one-year mortality rate was similar between the groups (21.7% vs. 17.9%, *p* = 0.493). There was also the improvement of NYHA functional class and sPAP reduction in both. This indicates that PMR could be a viable option for the treatment of patients with severe MR and ADHF [15].

A second paper on patients with MR looked at the impact of post-PMR change in left ventricular function on survival. The authors, led by Hagnäs et al., studied outcomes of 399 patients who underwent percutaneous edge-to-edge mitral valve repair for secondary MR and divided the cohort into three groups: unchanged (*n* = 318), improved (*n* = 40), and decreased (*n* = 41). For a median follow-up time of 2 years, following adjustment to confounders, decreased postprocedural left ventricular ejection fraction (LVEF) was associated with an increased risk of death (adjusted HR 2.05, *p* = 0.004), whereas increased postprocedural LVEF was associated with a reduced risk of death (adjusted HR 0.47, *p* = 0.024) compared to the unchanged LVEF group [16].

For patients with TR, significant heterogeneity exists regarding the leaflet number and shape, right ventricular anatomy, proximity of the annulus to the right coronary artery, and other parameters related to the ventricle and valve. Comprehensive imaging is expected to play a key role in the planning and periprocedural assistance of percutaneous TR repair [17,18]. In the TRIMA (Tricuspid Regurgitation Imaging) study, Cammalleri et al. assessed the geometrical characteristics of the tricuspid valve complex using novel computed tomography (CT) parameters [19]. In this small prospective study, patients with severe TR underwent a cardiac CT study dedicated to the right chambers. Variables obtained included: septal-lateral and antero-posterior diameters, tenting height, anatomical regurgitant orifice area annulus area and perimeter, distance between commissures and between the valve’s centroid and commissures, and angles between centroid and commissures. A significant phasic variability during the cardiac cycle existed for all variables except for eccentricity, angles, distance between the postero-septal and antero-posterior commissure, and distance between the centroid and antero-posterior commissure. There was a significant relationship between the tricuspid valve annulus area and novel annular parameters. Further studies are expected to follow that should shed light on the complexity of the tricuspid valve annular morphology, contributing to improvement in percutaneous TR therapeutics.

Currently, the predominant percutaneous TR therapy is edge-to-edge repair using the MitraClip/TriClip Transcatheter Tricuspid Valve Repair Systems (Abbott, Santa Clara, CA, USA) and the PASCAL transcatheter valve repair system (Edwards Lifesciences, Irvine, CA, USA). Medical centers throughout the world are reporting their initial edge-to-edge repair experience for TR. In our Special Issue, Cepas-Guillen et al. report their initial experience with edge-to-edge repair using the MitraClip and TriClip systems, assessing the efficacy and safety in the first consecutive 28 patients with severe TR. Only one patient experienced a procedural complication (femoral pseudoaneurysm). At three-month follow-up, 83% of patients were in NYHA I or II (18% baseline vs. 83% at 3-month follow-up; *p* < 0.001). Residual TR was ≤2 in 79% of patients (paired *p* < 0.001) [20].

In addition, the valve-in-valve (ViV) technique is becoming a viable percutaneous alternative for the treatment of bioprosthetic structural valve deterioration (SVD) in the tricuspid position. Our Special Issue includes two articles discussing this clinical matter. In a case report and review by Montenegro da Costa et al., they describe the implantation of a 32 mm MyVal transcatheter aortic valve device (Meril Life Sciences Pvt. Ltd., Vapi, Gujarat, India) in a 58-year-old female with a history of bioprosthetic valve implanted at the tricuspid position 27 years prior to the index event. The valve had deteriorated, resulting in severe tricuspid stenosis and mild TR. Treatment using the ViV technique was successfully performed, resulting in a reduction in the mean gradient to 3.3 mmHg [21]. From our center, Nili Schamroth Pravda et al. described the experience of the first 12 patients with tricuspid structural valve degeneration treated by the ViV technique [22]. All patients were treated with a balloon-expandable device. TR was ≥ moderate in 57.2% of patients at baseline, decreasing to 0% following the procedure. The mean trans-tricuspid valve gradients mildly decreased to 7.0 mmHg at one month following the procedure (*p* = 0.36). Mortality at one year was 8.0%. It thus seems that the tricuspid ViV procedure is feasible and safe, and certainly a less invasive treatment option (versus redo-open heart surgery) for patients with tricuspid valve degeneration.

Finally, Abdul-Jawad Altisent et al. describes the bicaval valve implantation (CAVI) technique in patients with severe TR [23]. The procedure is based on the heterotopic placement of a valve in the inferior vena cava (IVC) and another in the superior vena cava (SVC) at the cavo-atrial junctions. For this emerging method, there is a relatively scarce amount of data published regarding its efficacy and safety. Few initial first-in-man studies showed promising results, and four different CAVI approaches have been investigated: a non-dedicated device (e.g., using “over the shelf” balloon expandable valves with anchors) and three dedicated devices specifically designed for caval anatomy (e.g., TricValve^®^, Tricento^®^, and Trillium^TM^ systems). The author suggests that procedures with a CAVI-specific device are relatively simple and predictable, requiring fewer anatomical pre-requisites and interventional skills than other percutaneous tricuspid devices. However, the currently ongoing exploratory trials as well as future pivotal randomized studies versus optimal medical treatment and other therapeutic solutions will shed more light on the forecast of CAVI.

In summary, the field of percutaneous treatment of atrio-ventricular valves is gaining momentum as more evidence becomes available regarding the optimal non-invasive assessment of patients with MR and TR prior to the procedure, the best timing and selection of patients who could benefit from intervention, as well as their prognosis. New percutaneous technologies, and the experience gained from the clinical studies assessing them, will likely lead to improved techniques and a better understanding of the broad aspects of these complex valvular heart disease patients.
